# Mortality and guideline‐directed medical therapy in real‐world heart failure patients with reduced ejection fraction

**DOI:** 10.1002/clc.23664

**Published:** 2021-08-03

**Authors:** Peter A. McCullough, Hirsch S. Mehta, Colin M. Barker, Joanna Van Houten, Sarah Mollenkopf, Candace Gunnarsson, Michael Ryan, David P. Cork

**Affiliations:** ^1^ Texas A & M College of Medicine Dallas Texas USA; ^2^ San Diego Cardiac Center SHARP Healthcare San Diego California USA; ^3^ Department of Medicine, Division of Cardiology Vanderbilt University Medical Center Nashville Tennessee USA; ^4^ Edwards Lifesciences Irvine California USA; ^5^ Gunnarsson Consulting Jupiter Florida USA; ^6^ MPR Consulting Cincinnati Ohio USA; ^7^ Scripps Clinic La Jolla California USA

**Keywords:** goal directed medical therapy, heart failure with reduced ejection fraction, mortality, real‐world evidence

## Abstract

**Objective:**

To estimate the prevalence of guideline‐directed medical therapy (GDMT) in commercially insured US patients with heart failure with reduced ejection fraction (HFrEF) and examine the effect of GDMT on all‐cause mortality. GDMT for HFrEF includes pharmacologic therapies such as β‐blockers (BB), angiotensin‐converting enzyme inhibitors (ACE‐I), angiotensin receptor blockers (ARB), angiotensin receptor‐neprilysin (ARNI), mineralocorticoid receptor antagonists (MRA), and sodium‐glucose cotransporter inhibitors to reduce morbidity and mortality.

**Methods:**

Patients in the Optum Integrated File from 2007 to 2019Q3, ≥18 years, with history of HFrEF, were identified. Patients prescribed both a BB and either an ACE‐I, ARB, or ARNI during 6‐month post‐diagnosis were assigned to the GDMT cohort. All others were assigned to the not on GDMT cohort. The GDMT cohort was further classified by those patients with a record of prescription fills for both classes of medications concurrently (GDMT concurrent medication fills). Mortality at 2 years was assessed with a Cox regression model accounting for baseline demographics, comorbidities, and diuretic use.

**Results:**

This study identified 14 880 HFrEF patients, of which 70% had a record of GDMT, and 57% had a record of concurrent prescriptions. Patients in the not on GDMT cohort had 29% increased risk of mortality versus GDMT (hazard ratio 1.29; 95% CI (1.19–1.40); *p* < .0001). As a sensitivity analysis, the effect of patients not on GDMT compared to GDMT with concurrent medication fills was more pronounced, with a 37% increased mortality risk.

**Conclusion:**

In a real‐world population of HFrEF patients, inadequate GDMT confers a 29% excess mortality risk over the 2‐year follow‐up.

## 1 INTRODUCTION

Heart failure (HF) is a growing epidemic that affects approximately 23 million people worldwide and 6.2 million in the United States.^1^ It is associated with high rates of hospitalization, and 5‐year mortality after diagnosis is as high as 75%.^2^ The type and management of HF is increasingly characterized by ejection fraction (EF). Patients with an EF of 40% or less, accompanied by structural abnormalities in the left ventricle, are said to have reduced EF.^3^ These cases make up about half of all HF patients. Clinical guidelines for the treatment of HFrEF have been tested extensively with strong improvements in condition and include one medication from at least two classes.^4^ Either an angiotensin‐converting enzyme inhibitor (ACE‐I) or one of its alternatives, plus a beta blocker (BB) and in many cases, a mineralocorticoid receptor antagonist, constitute guideline‐directed medical therapy (GDMT) and are recommended in HFrEF.^3^ These medications have been demonstrated to significantly improve prognosis, reduce HF hospitalizations, and decrease the risk of cardiovascular death. Unfortunately, the multidrug regimen of GDMT is more difficult to implement and optimize outside of the setting of controlled clinical trials. Many HFrEF patients are not maintained on GDMT, which is potentially caused by gaps in clinical implementation, drug tolerability, and/or patient comorbidities. In this analysis, we consider such patient‐level factors in characterizing the uptake of and effect of GDMT among real‐world HFrEF patients in the United States. The purpose of our study is to establish the impact of GDMT (and the lack thereof) on overall mortality. Our hypotheses were (1) a substantial portion of real‐world HFrEF patients are not receiving GDMT; (2) those patients not on GDMT will have reduced survival over 1–2 years.

## 2 METHODS

### 2.1 Data source

All data used to perform this analysis were de‐identified and accessed in compliance with the Health Insurance Portability and Accountability Act. As a retrospective analysis of a de‐identified database, the research was exempt from IRB review under 45 CFR 46.101(b)(4). This study used data from the Optum integrated file, which contains data from the intersection of United Healthcare claims and Optum's electronic health records database.^5^ To be included in this integrated file, patients must be enrolled in United Healthcare insurance and have at least one hospital encounter in the electronic health records data in the window of data availability (2007–Q3 2019). The combination of claims and clinical data provides a comprehensive view of a patient's clinical interactions with the healthcare system. Optum data provides a continuum of treatment and cost information, such as medications by therapeutic area, provider notes with treatment rationale, and cost by procedure and condition. For this analysis, we utilized the electronic health record data to assess EF since these measurements would be recorded in the hospital setting. We utilized the claims data to assess comorbidities, drug utilization, and outcomes since this information is contained in the longitudinal United Healthcare payer database.

### 2.2 Inclusion/exclusion criteria

Patients in the Optum Integrated File from January 1, 2007 through September 30, 2019 with a record of HFrEF were identified by a series of patient selection queries. HFrEF was measured by either (1) a record of an EF of ≤40% found in the electronic health records portion of the Optum integrated database; or (2) diagnosis of systolic HF via ICD coding followed by at least one prescription fill for an angiotensin receptor neprilysin inhibitor (ARNI), which is almost exclusively used in patients with HFrEF. Patients were required to be 18 years of age or older and had 6 months of continuous health plan enrollment prior to their diagnoses of HFrEF as a means to identify comorbid conditions. Patients were also required to have 6 months of continuous enrollment following their HFrEF diagnosis in which to capture HF drug utilization. The initial 6 months after HFrEF diagnosis was considered a landmark period, during which drug regimens may be added to and titrated slowly according to recommendations.^6^

### 2.3 Cohort definitions

Patients with a prescription record from the claims database of both a BB and either ACE‐I, angiotensin receptor blocker (ARB), or ARNI during the 6‐month landmark period following their HFrEF diagnosis were considered to be on at least minimal GDMT and assigned to the GDMT cohort. Patients not meeting this criteria, regardless of whether they had a record of one class of medication or none, were assigned to the not on GDMT cohort. As a sensitivity analysis, the GDMT cohort was further classified by those patients having a record of prescription fills for both classes of medications (BB and ACE‐I/ARB/ARNI) concurrently at some point within the 6‐month landmark period; this subset was labeled GDMT with concurrent medication fills. A patient had to have at least 1 day of overlapping prescriptions with up to a 7 day gap in both classes.

### 2.4 Outcome of interest

The primary outcome was all‐cause mortality, measured up to 2 years from the end of the landmark period (6 months after a patient's diagnosis of HFrEF in which drug regimens were assessed).

### 2.5 Covariates of interest

The covariates for this analysis included patient demographics and comorbidities. Demographics considered included age, sex, race, insurance, and region. The Elixhauser Comorbidity Index, a validated set of 31 categories of comorbidities associated with mortality, was used to quantify patient baseline comorbidities. Each of the included comorbidities was identified using diagnosis codes that appeared in the 6‐month period before a patient's index diagnosis of HFrEF.

Since patients with concomitant diseases may be contraindicated for certain GDMT drugs, patients with a record of certain key comorbidities prior to their HFrEF diagnosis were separately flagged even though they were also represented in the comorbidity index. These chronic diseases included atrial fibrillation, complicated hypertension, chronic coronary artery disease, and chronic kidney disease. Finally, although diuretics are not part of the strict definition of GDMT, patients with a record of diuretics during the landmark period were also flagged.

### 2.6 Statistical analysis

The two main cohorts (GDMT, not on GDMT) were described by patient characteristics and comorbid conditions. Descriptive analytics were presented as mean and standard deviation for continuous variables or count and percentage for categorical variables. All‐cause mortality was estimated for the not on GDMT cohort versus the GDMT cohort using a Cox hazard model and adjusted for patient demographics and key comorbidities (atrial fibrillation, complicated hypertension, chronic coronary artery disease, chronic kidney disease), and whether they had a record of diuretic use. As a sensitivity analysis, the model was repeated for the subset of GDMT patients that had a record of concurrent medication fills as the comparison group. Hazard ratios (HR) and 95% confidence intervals (CI) were provided as measures of strength of association and precision, respectively.

## 3 RESULTS

**FIGURE 1:**
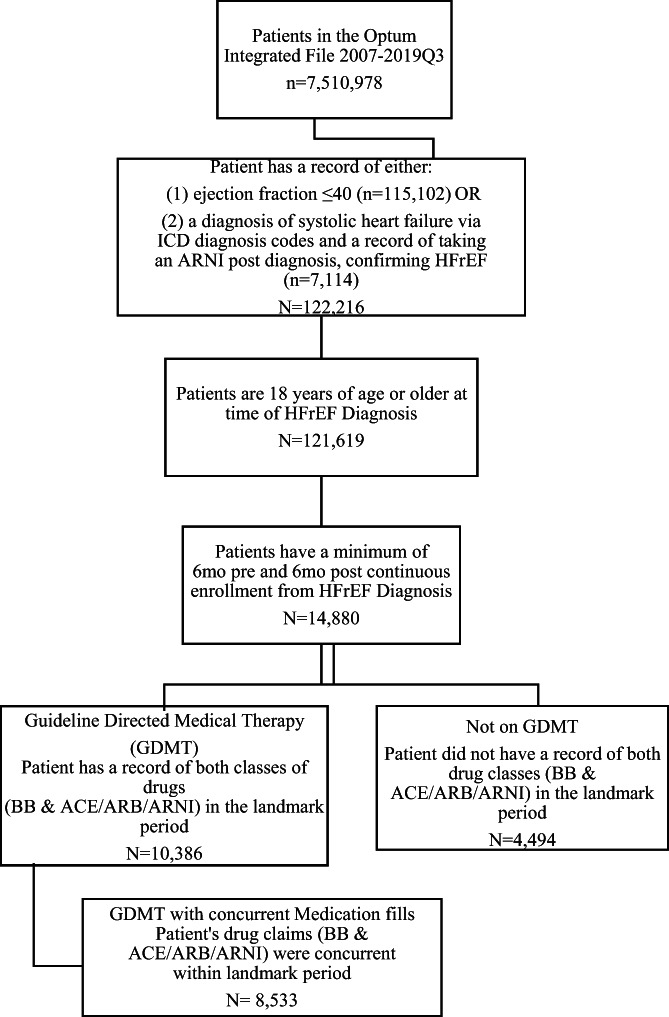
Patient attrition diagram. Patients in the Optum database were selected for inclusion in the analysis using the steps below. Eligible HFrEF patients were then classified as GDMT (medications from at least 2 classes), GDMT (concurrent medications from at least 2 classes), and not on GDMT. GDMT, guideline‐directed medical therapy

A total of 122 216 patients from 2007‐2019Q3 in the Optum Integrated File were identified as HFrEF by having either a record of an EF of ≤40% (n = 115 102, 94.2%) or a record of a diagnosis code for systolic HF and a record of at least one medication fill for an ARNI following the systolic diagnosis (n = 7114, 5.8%) (Figure 1). Patients were required to be adults, 18 years of age or older at the time of their HFrEF diagnosis, which reduced the sample to 121 619. In order to measure comorbidities prior to HFrEF diagnosis and to assess pharmacologic response after, 6 months of continuous enrollment both before and after HFrEF diagnosis were required in Optum's claims portion of the integrated database; this significantly reduced our sample size from 121 619 to 14 880. After inclusion criteria were applied, the final cohort assignments were as follows: GMDT cohort 10 386 (69.8%), versus not on GDMT cohort 4494 (30.2%). When the GDMT cohort was further restricted to requiring concurrent medication fills, the GDMT cohort of 10 386 was reduced by 17.8% to 8533 patients.

Patient demographics (age, sex, region, race, and insurance type), comorbidity index, and presence of atrial fibrillation, complicated hypertension, coronary artery disease, chronic kidney disease, and use of diuretics during the landmark period are shown in Table 1. Demographic variables were fairly similar across the two main cohorts with average age ranging from 68 to 71 years old. The highest rates of patients were male (61%+), Caucasian (85%+), from the Midwest (over 43%+) and insured with Medicare (69%+). The mean Elixhauser comorbidity score was higher for the GDMT cohort (6.3) compared to Not on GDMT (5.8). When comparing individual comorbid conditions at baseline, there were higher rates of complicated hypertension (37.9% vs. 29.3%) and coronary artery disease (74.9% vs. 62.9%) in the GDMT cohort. However, there were higher rates of atrial fibrillation (40.9% vs. 30.9%) in the not on GDMT cohort as compared to the GDMT cohort, and there were similar rates of chronic kidney disease between the two groups.

**TABLE 1:** Patient characteristics

	GDMT	GDMT with concurrent medication fills	Not on GDMT control group
Number of patients	10 386	8533	4494
Age mean (SD)	69.2 (11.9)	68.6 (11.9)	70.9 (12.8)
Gender			
Female	3538 (34.1%)	2936 (34.4%)	1717 (38.2%)
Male	6844 (65.9%)	5595 (65.6%)	2774 (61.8%)
Race			
Asian	104 (1.1%)	92 (1.1%)	44 (1.0%)
Black	1295 (13.1%)	1112 (13.8%)	376 (8.8%)
Caucasian	8476 (85.8%)	6882 (85.1%)	3877 (90.2%)
Region			
Midwest	4514 (44.6%)	3591 (43.2%)	1905 (43.9%)
Northeast	974 (9.6%)	816 (9.8%)	399 (9.2%)
South	3160 (31.2%)	2695 (32.4%)	1223 (28.2%)
West	1472 (14.6%)	1218 (14.6%)	808 (18.6%)
Payor			
Commercial	3108 (29.9%)	2643 (31.0%)	1220 (27.2%)
Medicare	7278 (70.1%)	5890 (69.0%)	3274 (72.9%)
Elixhauser comorbidity index mean (SD)	6.3 (3.1)	6.3 (3.0)	5.8 (3.3)
Atrial fibrillation in pre period	4100 (30.9%)	3247 (38.05%)	1837 (40.9%)
Complicated hypertension in pre period^a^	3936 (37.9%)	3187 (37.35%)	1316 (29.3%)
Coronary artery disease in pre period	7789 (74.9%)	6370 (74.65%)	2829 (62.9%)
Chronic kidney disease in pre period	2677 (25.8%)	2115 (24.79%)	1250 (27.8%)
Concurrent diuretics in landmark period	7860 (75.68)	6413 (75.16%)	2419 (53.83%)

Abbreviations: GDMT, guideline‐directed medical therapy; pre period, 6 months prior to HFrEF diagnosis; Landmark period, 6 months following HFrEF diagnosis.

^a^ Complicated hypertension = hypertensive heart disease with heart failure (code I11 in International Classification of Diseases, version 10.).

**FIGURE 2:**
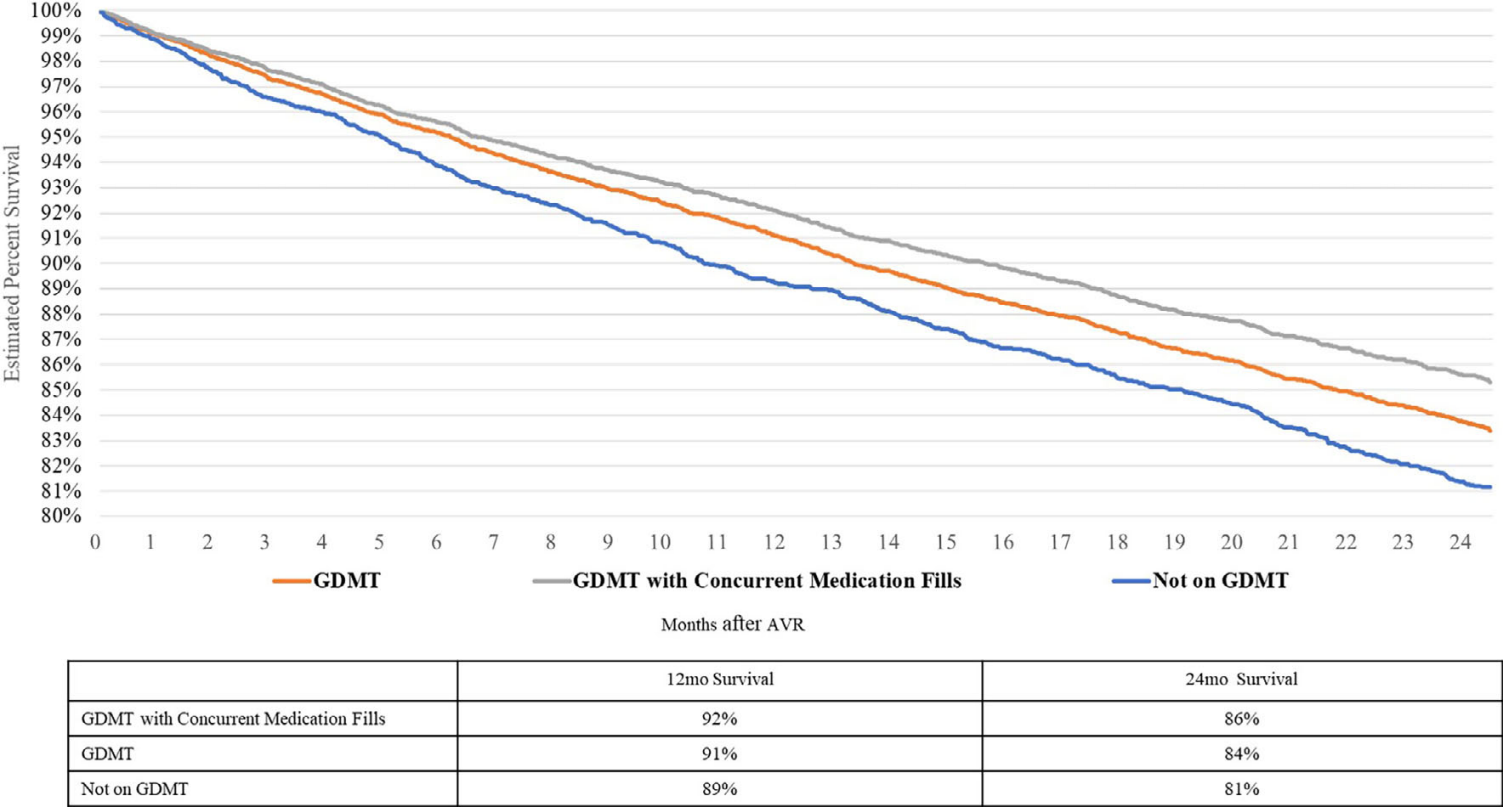
Central figure—survival curves from Cox regression models. The survival curves shown are generated from multivariable Cox proportional hazard models and show a higher 2‐year survival rate for patients on guideline‐directed medical therapy (GDMT), particularly for patients on concurrent medication: 86%, GDMT 84%, versus 81% not on GDMT. In the model, patients not on GDMT are 1.29 (compared to GDMT) and 1.37 (compared to GDMT with concurrent medication fills) times more likely to die at any point in time within the 2‐year post‐landmark period. This equates to an excess mortality risk of 29% and 37%, respectively

Table 2 reports HRs for all‐cause mortality from the Cox regression models along with all model covariates, while Figure 2 displays the 2‐year survival for each cohort of interest from the results of the Cox regression. The survival curves show a higher 2‐year survival rate for patients on GDMT, particularly the more strict definition with concurrent medication fills (86% GDMT with concurrent medication fills, 84% GDMT, vs. 81% Not on GDMT). In the multivariable model, patients not on GDMT are 1.29 (compared to GDMT) and 1.37 (compared to GDMT with concurrent medication fills) times more likely to die at any point in time within the 2‐year post‐landmark period. This equates to an excess mortality risk of 29% and 37%, respectively.

**TABLE 2:** Annualized mortality risk by GDMT: multivariable regression

	Not on GDMT versus GDMT			Not on GDMT versus GDMT with concurrent medication fills		
	Hazard ratio	95% confidence interval	*p* value	Hazard ratio	95% confidence interval	*p* value
Not on GDMT versus GDMT or GDMT with concurrent medication fills	1.29	1.19–1.40	<.0001	1.37	1.26–1.49	<.0001
Age (per year increment)	1.03	1.03–1.04	<.0001	1.03	1.03–1.04	<.0001
Sex						
Male	1.11	1.02–1.20	.0102	1.15	1.05–1.25	.0015
Female	*1.0 (ref)*	*1.0 (ref)*		*1.0 (ref)*	*1.0 (ref)*	
Race						
Black	2.84	1.51–5.33	.0012	2.60	1.38–4.89	.0031
Caucasian	2.62	1.41–4.89	.0023	2.40	1.29–4.46	.0059
Asian	*1.0 (ref)*	*1.0 (ref)*		*1.0 (ref)*	*1.0 (ref)*	
Region						
Northeast	0.75	0.66–0.86	<.0001	0.76	0.66–0.87	.0001
South	0.65	0.60–0.72	<.0001	0.64	0.58–0.71	<.0001
West	0.62	0.55–0.69	<.0001	0.61	0.55–0.69	<.0001
Midwest	*1.0 (ref)*	*1.0 (ref)*		*1.0 (ref)*	*1.0 (ref)*	
Record of diuretics (none vs. at least one diuretic prescription fill)	1.86	1.69–2.04	<.0001	1.86	1.68–2.06	<.0001
Atrial fibrillation in pre period	1.16	1.07–1.25	.0001	1.17	1.08–1.27	.0002
Complicated hypertension in pre period	0.92	0.83–1.01	.0724	0.92	0.83–1.03	.1429
Coronary artery disease in pre period	1.30	1.19–1.42	<.0001	1.33	1.20–1.46	<.0001
Chronic kidney disease in pre period	1.30	1.18–1.44	<.0001	1.28	1.15–1.43	<.0001

Abbreviations: GDMT, guideline‐directed medical therapy; *ref*, reference group; pre period, 6 months prior to HFrEF diagnosis.

## 4 DISCUSSION

This retrospective analysis found that patients not managed on GDMT had a significantly increased risk of 2‐year mortality compared to patients on GDMT. We found 2‐year mortality to be approximately 20% in HFrEF patients, similar to results published in the PARADIGM‐HF trial, which demonstrated a rate slightly less than 20%.^7^ Other studies have found higher mortality rates at 2 years in this population, such as the EVEREST trial with a rate of 30% and the GWTG‐HF study, in which 2‐year mortality approached 50%.^2^, ^8^ In the present analysis, patients not on GDMT had a 29% increased risk of all‐cause mortality in the adjusted findings accounting for patient demographics, key comorbidities, and presence of diuretics (Table 2). Outcomes diverged even further in the sensitivity analysis when the comparison of GDMT with concurrent medication fills versus not on GDMT, with the latter having an increased mortality risk of 37%. In our study approximately 70% of patients had a record of GDMT, so in a real‐world setting around 30% of patients are not receiving pharmaceutical treatment according to guidelines for a host of reasons.

### 4.1 Clinical context

The combined pharmacologic therapies that form the cornerstone of clinical guidelines for HF and New York Heart Association classifications have been proven to dramatically improve health outcomes, but surprisingly few patients are actually receiving the recommended treatments. Deschaseaux, et al.^9^ found adherence rates of 0.65–0.87 (as measured by proportion of days covered), and noted that 29% of patients never received HF medication in the index period following their HF diagnosis (this rate is similar to the rate of patients not on GDMT in our analysis). When criteria for GDMT adherence are expanded to include sufficient dosage and the use of an MRA, the proportion of patients receiving optimal treatments drops to fewer than 1%, as found by the CHAMP‐HF registry.^10^ In that large‐scale prospective trial, approximately 26% of patients were not prescribed an ACE inhibitor, ARB or ARNI, more than a third of patients were not prescribed a BB, and over half were not on an MRA.

Drug tolerability is a major factor in adherence to medication regimens. Deschaseaux and colleagues reported that many of the treatment changes in the months following a HF hospitalization involved a de‐escalation of therapy.^9^ Considering the status of HF hospitalizations as a “transition point” after which health outcomes tend to see a steep decline and fewer clinical markers or subcategories (such as EF) provide much prognostic significance, it is all the more interesting that the aggressiveness of treatment often goes in the opposite direction it needs to in order to slow cardiovascular decline.^11^ Furthermore, due to the interaction of GDMT drugs with other comorbidities, as well as the medications used to treat them, some patients have to be taken off GDMT, or are contraindicated from commencing it in the first place.^12^, ^13^

### 4.2 Strengths

A strength of this study is that it characterizes HF by EF, which many large retrospective studies cannot due to the limited types of data available in an administrative claims database. In this analysis, claims data are combined with electronic health records, and adjustments were made for patient characteristics known to affect tolerability and mortality. The broad selection criteria employed in the use of a large data set also helped preserve generalizability.

### 4.3 Limitations

As with all studies that rely on automated sources of data, it is possible that parameters such as billing codes could be biased and proxies could fail to capture certain factors difficult to ascertain from the available clinical data. Our use of a proxy (prescription of ARNI) for reduced EF as an alternative to echocardiogram may skew our cohorts toward a higher percentage of patients on GDMT, since ARNI is one qualifying component of the drug regimen. However, 94.2% of our HFrEF population of interest had a record of an echocardiogram result of an EF of ≤40, and only a small percentage, 5.8%, relied on ICD coding and a record of taking an ARNI.

We were unable to adjust for vital signs, laboratory data, region, and other potential confounding measures due to the lack of clinical parameters in this real‐world data source. Consequently, we were unable to differentiate patients that are not eligible for GDMT (due to heart rate, blood pressure, renal function, etc.), also recognizing that inability to tolerate GDMT portends worse outcomes. However, we adjusted for baseline differences in many comorbid conditions that may deem patients ineligible for GDMT, e.g. chronic kidney disease.

We required continuous enrollment both 6 months prior to and after HFrEF diagnosis. This was necessary to ensure data collection on baseline comorbidities and medication regimens, but may induce bias toward healthier patients (i.e., those with early death during the first 6 months would not be included in the analysis). We expect that the relationship between GDMT and all‐cause mortality should be preserved, as the same continuous enrollment periods were applied to each cohort. Patient adherence may also be inadequately captured by the use of prescription fills to determine medication status, as not everyone who fills their prescription is taking the drugs as intended. Given the patients represented in our analysis, results are generalizable only to the commercially insured patient population.

Finally, we acknowledge that in many cases, a mineralocorticoid receptor antagonist, constitutes GDMT and is recommended in HFrEF. However, our goal was to cast the broadest net possible to capture GDMT patients based on the minimum requirement for GDMT, which is the combination of ACEI/ARB/ARNI and BB. Due to the low tolerance of mineralocorticoid receptor antagonists, the inclusion of these drugs as a requirement for GDMT would have limited our treatment group substantially.

## 5 CONCLUSION

In a real‐world population setting, 30%–42% of HFrEF patients were not optimally managed on GDMT; the absence of GDMT was independently associated with increased mortality. For every year of inadequate GDMT, there was at least a 29% increased risk of mortality. There are varieties of barriers to GDMT for HFrEF patients, especially given the high rate of comorbidities for this demographic, but improving clinical implementation of combined pharmacologic therapies and augmenting them with surgeries and new devices as needed is an effective path for improving health outcomes.

## 6 CLINICAL PERSPECTIVES

### 6.1 Competencies in medical knowledge

The failure to treat patients eligible for GDMT with the full extent of pharmacologic therapy in routine clinical practice contributes to the high mortality rate of HF, an increasingly prevalent condition. Patient nonadherence, medication intolerance, and contraindications compound this problem. Improved attention to state‐of‐the‐art care, together with broadening treatment options, would help reduce preventable deaths and improve quality of life for cardiovascular patients.

### 6.2 Translational outlook

Investigating causes behind patient nonadherence is an important avenue for future research, and the development of more nonpharmacologic treatment options may aid in the treatment of patients who are intolerant of or unresponsive to traditional GDMT.

## CONFLICT OF INTEREST

All authors contributed significantly to the work and meet the criteria for authorship; all have read and approved the manuscript. Relevant industry relationships and financial interests are as follows: Colin M. Barker, David P. Cork, Peter A. McCullough, Hirsch S. Mehta, Michael Ryan, and Candace Gunnarsson have consulting relationships with Edwards Lifesciences. Dr. Barker is an advisory board member for Medtronic and Boston Scientific. Dr. Cork has a consulting relationship with Abbott Laboratories and participates in a speaker's bureau for Boston Scientific. Dr. Mehta has a consulting relationship with Abbott Laboratories, Boston Scientific and participates in a speaker's bureau for Actelion Pharmaceuticals, Bayer Healthcare Pharmaceuticals, and Bristol‐Myers Squibb Company. Joanna Van Houten and Sarah Mollenkopf, are employees of Edwards Lifesciences, the study sponsor. Standard COI forms have been filled out and provided.

## Data Availability

The data that support the findings of this study are available from Optum. Restrictions apply to the availability of these data, which were used under license for this study. Data are available at request of corresponding author and with the permission of Optum.
